# The Story of the Hospitals

**Published:** 1886-12-04

**Authors:** 


### The Story of the Hospitals.

(Continued from page 110.)

The Out-Patients.

I shall have, in the course of my "Story of the Hospitals," a great deal to say with regard to the various systems which prevail as to the management of out-patients, and in particular as to the excellent scheme of reform which has, since 1884, been adopted at the London Hospital. But this is a very wide question, on which I will forbear entering to-day. The out-patients' department of a great London Hospital supplies inexhaustible materials for the study of every phase of human life as it is to be seen among the poor and labouring classes. The young and the old, the gentle and the rude, the timid and the hardened, the educated and the illiterate, are here thrown together to await the surgeon's skill or the dispenser's compound. Poverty is about the only attribute common to them all, for it is soon evident to the observer that, despite rough and jaded apparel, there are natures as divergent as the poles. Some of the frequenters of the out-patients' department of the London Hospital walk in with a bold and self-assertive air, evidently the outcome of matured acquaintance; while others betray, by nervous step and furtive glance, that the whole scene is new to them. The attendant in the Waiting Hall marshals and directs the applicants with quick and decisive orders which allow of no appeal. "Take your seat, man, next to the last lady on the right," is an oft-repeated command. "Are you an accident?" he inquires of another; "then take your place next to the last man and answer your name when it's called." Why "lady" in one instance and "man" in the other, I desired to learn, but my curiosity has not yet been appeased.

The Artist's Model.

I notice among the group of applicants a young girl of about eighteen. Her face attracted my attention, for there was a saddened beauty about it. Her left hand was in a sling, and she appeared to me to be in pain. She sat, quiet and pensive, among some women of the roughest class. On speaking to her, I learnt that the limb was not broken, as I had imagined from the bandaging, but that there was a painful gathering on the hand. She was dressed with exceeding neatness, and answered my inquiries in a subdued voice. "I have never," she said, "been in an hospital before: I have tried to help myself, but my hand seemed to get worse, and I felt forced to come here. I do hope they will do me good, for I have a mother and a little brother to support." As she spoke these words, tears rose to her eyes, and it was only with some effort that she could stifle the emotion within her. Her story interested me, and I ventured to put a few further questions to her. "Until recently, sir," she continued, "I used to sit to artists, but I don't sit now: I help mother at the dressmaking, but, with my hand bad I cannot work the machine, and mother's sight is too bad to do it. This, sir, is the cause of my anxiety, for if we fail to pay the rent, our little home is gone."

Her History.

"An artist's model," I mused. That pretty face, which sadness had invested with a peculiar charm, had been the object of many a painter's study. "I was just fifteen, sir," she continued, "when I first went out to sit: it was at the studio of Mr. —— at Chelsea. He introduced me to some of his artist-friends, and, as it happened to be just the season before the opening of the Academy, I was soon sitting almost every day. I made a lot of money—three pounds a week sometimes, by sitting on Sundays as well as week-days." "On Sundays?" I queried. "Oh, yes, sir, many of the artists paint on Sundays, which are often very busy days with girls of the class I was then entering. I've sat at Calderon's School at St. John's Wood, at Herkomer's at Bushy, at South Kensington, and at private schools, but I never liked the calling. Among artists, as among other classes, there are some who are not gentlemen. My father was living then, but, though he knew I went to the studios, he did not know I sat for the 'figure,' or, sir, he would never have allowed it. Mother did not know it, either, at first, but I told her afterwards. When the work fell off this summer, I determined to give up sitting, and to help mother with dressmaking—and now, sir, to think I can't do that! Oh, I do hope they will be able to do me good." Here her name was called and our conversation closed.

I Meet Her.

I chanced to meet Miss H.—artists' models are always "Misses"—in the Whitechapel Road some days after the interview at the London Hospital. Her hand, she told me, was then quite well again. But yet another trouble: her mother had fallen sick. "But sir," she added, "you know I'm all right now, and I can work, and I know that if I'm good to mother—and I mean to be—I shall get her right again."

> "One sorrow never comes but brings an heir,
> That may succeed as his inheritor."

When the Prince of Wales went to Whitechapel.

Last midsummer, the Prince of Wales went to Whitechapel to lay the foundation stone of the People's Palace. One would hardly have thought that the royal progress to the East-end, accompanied as it was with every manifestation of orderly loyalty and good behaviour on the part of the denizens of Whitechapel, would in any way have materially affected the London Hospital. A few isolated cases and slight injuries might have been expected, but considering the peaceful character of the demonstration accorded to the Prince of Wales, it would hardly have been anticipated that there would have been much need for doctors' services. Quite a mistake. Within the walls of the Hospital on the day when the East-enders turned out in their masses to welcome their future king with all the decorum of loyal citizens, no less than one hundred casualties in connection with the occasion were attended to at the London Hospital. Of these, eight were what the doctors call "bad cases," seventeen were those of injuries sufficiently serious to need "out-patient" treatment, while the balance were minor mishaps attended to on the spot. It is lucky for the London Hospital that royalty does not go to Whitechapel every day.

